# A foundation for complex oxide electronics -low temperature perovskite epitaxy

**DOI:** 10.1038/s41467-020-16654-2

**Published:** 2020-06-08

**Authors:** Henrik H. Sønsteby, Erik Skaar, Øystein S. Fjellvåg, Jon E. Bratvold, Helmer Fjellvåg, Ola Nilsen

**Affiliations:** 1 Department of Chemistry, Center for Materials Science and Nanotechnology, University of Oslo, Blindern, 0315 Oslo, Norway; 2 Department for Neutron Materials Characterization, Institute for Energy Technology, 2007 Kjeller, Norway

**Keywords:** Electronic materials, Materials for devices, Electronic devices

## Abstract

As traditional silicon technology is moving fast towards its fundamental limits, all-oxide electronics is emerging as a challenger offering principally different electronic behavior and switching mechanisms. This technology can be utilized to fabricate devices with enhanced and exotic functionality. One of the challenges for integration of complex oxides in electronics is the availability of appreciable low-temperature synthesis routes. Herein we provide a fundamental extension of the materials toolbox for oxide electronics by reporting a facile route for deposition of highly electrically conductive thin films of LaNiO_3_ by atomic layer deposition at low temperatures. The films grow epitaxial on SrTiO_3_ and LaAlO_3_ as deposited at 225 °C, with no annealing required to obtain the attractive electronic properties. The films exhibit resistivity below 100 µΩ cm with carrier densities as high as 3.6 · 10^22^ cm^−3^. This marks an important step in the realization of all-oxide electronics for emerging technological devices.

## Abstract (web summary)

Designing complex oxides with appreciable low-temperature synthesis remains a challenge. Here, the authors demonstrate an atomic layer deposition process for LaNiO_3_ to enable epitaxial thin films on LaAlO_3_ and SrTiO_3_ substrates deposited at 225 °C, with no annealing required to achieve good electronic properties.

## Introduction

Traditional silicon transistor technology is on the verge of reaching its fundamental limits, and nearly 60 years after the postulation of Moore’s law the tech industry is now struggling to maintain continuous evolution in transistor density and computing power. Oxide electronics is considered a viable future contender to Si-based circuitry. Architectures utilizing the plethora of functional properties offered by complex oxides are already important in, e.g., optoelectronics, magnetoelectrics, spintronics and thermoelectrics^1^. Significant research effort is also currently put into the development of complex oxide field effect transistors (COFETs). Armed with fundamentally different switching mechanisms as compared to e.g. silicon-based *MOS*FETs, the COFETs will offer high-density electron systems that can be switched by lower potentials or different perturbing fields than what is applied in current technology^2–5^. These novelties are proposed to lead to more energy conserving and faster devices with less heat dissipation due to lower internal resistance: Increasingly important in the search of greener technology.

One of the most notable advantages of complex oxide electronics is the possibility to integrate several functional properties via epitaxy between two or more structurally similar compounds. In addition, the interfaces created between the employed structures are also often found to exhibit novel functional properties themselves^6–8^.

Complex oxides with the perovskite structure are ideal in terms of epitaxial integration of multiple layers. They are typically close to cubic with pseudocubic lattice parameters in the vicinity of 4 Å, offering the possibility of epitaxial interfaces. Compounds within or related to this structure type include ferromagnetic (e.g. (La,Sr)MnO_3_), ferroelectric (e.g. Pb(Zr,Ti)O_3_ and (K,Na)NbO_3_), high-κ dielectric (e.g. SrTiO_3_ and LaAlO_3_), superconducting (e.g. YBa_2_Cu_3_O_7−*δ*_) and multiferroic (e.g. BiFeO_3_) materials.

Rare-earth nickelates (*RE*NiO_3_, *RE* = trivalent rare-earth) have seen extensive research interest over the last few decades. This is for a large part due to a sharp metal-to-insulator transition (MIT) that is correlated to the Ni−O−Ni bond angle, which varies with the size of the rare-earth cation. The Ni−O−Ni bond angle determines the O_2p_–Ni_3d_ orbital overlap, with bands widening when the angle is large (i.e. large orbital overlap)^9,10^. The underlying structural and physical mechanisms behind the MIT is previously extensively reviewed^11–14^. Lanthanum nickelate, LaNiO_3_ (LNO), is a special member of the *RE*NiO_3_ group. The larger La^3+^ cation facilitates a bond angle that favors the formation of a metallic rhombohedral structure (*R*-3*c*) even at absolute zero^9,14^.

As a result, LNO remains a metal at all temperatures, and conducts electrons as such. Resistivity in single crystals is reported to be ~10^−4^ Ω cm at room temperature, with a temperature dependence following $\rho = \rho _0 + AT^n$ (*n* ≈ 2)^15,16^. The resistivity and metallic behavior can, however, be tuned by strain engineering. In-plane compression (e.g. on LaAlO_3_ (100)_pc_) leads to less orbital overlap and more narrow bands, while in-plane tensile strain (e.g. on SrTiO_3_ (100)) leads to wider bands.

Electrically conducting perovskite oxides are rare, and hence LNO is thought to play a role in a wide variety of applications where electronic conductivity is required^17–21^. Device integration requires LNO to take form as a thin solid layer, preferably epitaxially connected to a substrate. A technologically interesting example is LNO epitaxially integrated with SrTiO_3_ (STO), effectively making a perovskite oxide stack with a conducting electrode and a high-κ dielectric. This can enable the use of STO as a gate oxide in future transistors. Even further down the path this could become part of a fully integrated perovskite oxide electronics system, enabling faster and more energy-efficient electronic devices, e.g. COFETs.

The need for epitaxial integration is, however, also the main caveat for implementation of complex oxides in consumer electronic devices. Techniques used for the deposition of epitaxial complex oxide systems include molecular beam epitaxy and pulsed laser deposition, neither of which is straightforward to implement in a production line due to high-temperature, high-vacuum and limited area synthesis^22,23^. Chemical techniques like chemical vapor deposition (CVD) are also employed, but the resulting films require post-annealing at >600 °C to obtain epitaxy^24,25^. This effectively hampers its applicability in monolithic device integration.

As a result of this, low temperature-, low vacuum epitaxy of complex oxides on large areas is highly anticipated. The goal is simple: To develop a toolbox of functional materials that can be epitaxially integrated under conditions that are feasible for monolithic device integration. In addition, with increasing geometrical complexity of novel device architectures, synthesis technique that allows strong conformality control on high-aspect ratio substrates becomes more important.

Atomic layer deposition (ALD) is a derivative of CVD where the different precursors are separated as individual pulses in either time or space^26^. This enables self-limiting saturating growth with sub-monolayer thickness control. ALD does not require line-of-sight, enabling deposition on high-aspect ratio and/or hollow substrates, which is important in novel device architectures. Although ALD traditionally have been used to deposit binary oxides, heavier chalcogenides and halides, there has been an increase in reports of functional complex oxides over the last 10 years^27–29^.

Previous studies of ALD of LNO have resulted in amorphous films as deposited^30,31^. These films have always required post-annealing to convert into crystalline/epitaxial films, with a lowest reported annealing temperature of 750 °C.

In this study, we report on the use of an alternative precursor chemistry enabling as-deposited epitaxial LNO at 225 °C on a range of substrates and orientations. No post-annealing is required to obtain metallicity. The films exhibit long-range epitaxy as shown by scanning transmission electron microscopy (STEM) and reciprocal space mapping (RSM) by X-ray diffraction (XRD). The films conduct electrons with bulk-like resistivity and high carrier concentrations characterized by four-point probe and Hall measurements. The temperature dependence of the resistivity reveals that the films behave as metals at all temperatures, following a power law pointing towards Fermi liquid behavior. This novel low-temperature epitaxy of LNO creates a foundation for realization of a range of oxide-based devices where a highly electrically conductive perovskite template material is required, i.e. in COFETs. It extends the toolbox of available low-temperature deposited epitaxial complex oxides with appreciable functional properties.

## Results

### Atomic layer deposition process development

Multi-cation ALD is traditionally carried out by sequentially employing processes for binary oxides. In the case of LNO, this can be done by combining the La_2_O_3_ (La(thd)_3_ + O_3_) and NiO (Ni(acac)_2_ + O_3_) processes. Note that dry Ni(acac)_2_ is known to sublimate in the form of an octahedrally coordinated trimer, [Ni(acac)_2_]_3_. In this trimer, Ni−O−Ni bonds of similar length to those found in LaNiO_3_ are already present, and this is believed to be of high importance with respect to epitaxial growth. An important feature of the process is that both binary recipes use ozone as the oxidant. This is especially important in the case of La-containing depositions, as La is prone to form La(OH)_3_ upon reaction with water, effectively working as a water reservoir which can be detrimental to controlled growth. Also note that nickel is nominally in oxidation state 3+ in LNO, and that a strong oxidant is required to obtain this state.

The La(thd)_3_ + O_3_ process is known to exhibit controlled growth in the 200–350 °C temperature range, limited downwards by its applicable sublimation temperature (185 °C) and upwards by thermal decomposition of the precursor^32^. The Ni(acac)_2_ + O_3_ process has a similar low-temperature limit, but Ni(acac)_2_ starts to decompose already at around 300 °C ^33^. To minimize the thermal budget, but still facilitate controlled growth and a temperature gradient from the precursor source to the reaction chamber, we chose to employ a reactor temperature of 225 °C for the deposition of the La-Ni-O-system.

As both the size and reaction mechanism for the two cation precursors are different, resulting in a different GPC for the two processes, we expect that a 1:1 pulsed ratio of the two binary processes does not result in an equiatomic mixture of La and Ni. To understand the relationship between the pulsed and deposited cation ratios, we mapped the cation ratio space by employing a variable La(thd)_3_ + O_3_ to Ni(acac)_2_ + O_3_ ratio and measuring the resulting cation stoichiometry by X-ray fluorescence (XRF) (Supplementary Fig. 1). A 5:2 pulsed ratio between La(thd)_3_ + O_3_ and Ni(acac)_2_ + O_3_ is found to result in films with a 1:1 cation ratio in the deposited films. The effective ALD-recipe used for the remainder of the study is thus:

1 $$5 \times \left( {{\mathrm{La}}\left( {{\mathrm{thd}}} \right)_3 \, + \, {\mathrm{{O}}}_3} \right) + 2 \times \left( {{\mathrm{Ni}}\left( {{\mathrm{acac}}} \right)_2 \, + \, {\mathrm{O}}_3} \right).$$

This process facilitates a GPC of ~0.31 Å cycle^−1^, where a cycle is considered to be one cation precursor pulse followed by one pulse of ozone. Self-limiting behavior was confirmed by employing variable pulse- and purge durations within the complex process (Supplementary Fig. 2).

### Structural analysis by X-ray diffraction

Thin films of ~30 nm were deposited on a variety of single crystal substrates to determine the structure and functionality of LNO at variable strain. The films were amorphous as deposited on Si (100) (Supplementary Fig. 3), but is remarkably single-oriented crystalline as deposited on STO (100/110/111) and LAO (100/110/111) (Supplementary Fig. 4). This is an unexpected result, as multi-cation ALD films are very often amorphous and in need of post annealing to become crystalline due to the low deposition temperatures. Similar as-deposited epitaxy is recently shown for ferromagnetic NiFe_2_O_4_ and antiferromagnetic NiTiO_3_ as well as for LaMnO_3_ ^34–36^.

**Fig. 1:**
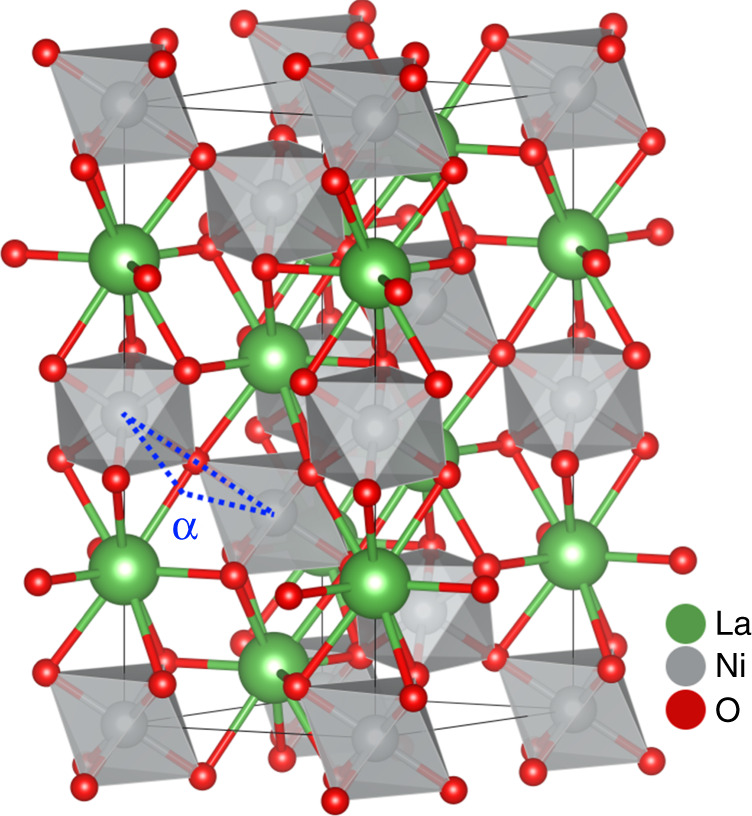
Crystallographic structure of LaNiO_3_. Octahedron configuration in bulk LaNiO_3_ showing the Ni−O−Ni bond angle *α* (165.2°) that facilitates the rhombohedral (R-3c) structure. Nickel ions (green) are centered in the gray octahedra, surrounded by oxygen (red).The tensile strain on STO (100) substrates leads to the bond angle increasing, creating a larger orbital overlap and wider bands, and thus a lower electrical resistance. On LAO (100) the bond angle decreases, leading to a smaller orbital overlap and more narrow bands, and thus a higher electrical resistance.

LNO (100) is the most technologically interesting orientation for oxide electronics. Thus, in the remainder of the article, we focus on the STO(100)||LNO(100)_pc_ and LAO(100)_pc_||LNO(100)_pc_ systems (pc = pseudocubic). Considering the bulk pseudocubic lattice parameter of LNO to be 3.84 Å, LNO will observe a theoretical 1.6% tensile strain on STO (100) and 1.3% compressive strain on pseudocubic LAO (100)_pc_. This is previously shown to be important for the in-plane conductivity of the material, since the Ni−O−Ni bond angle (and thus the orbital overlap) is dependent on the lattice parameter (Fig. 1)^9^.

**Fig. 2:**
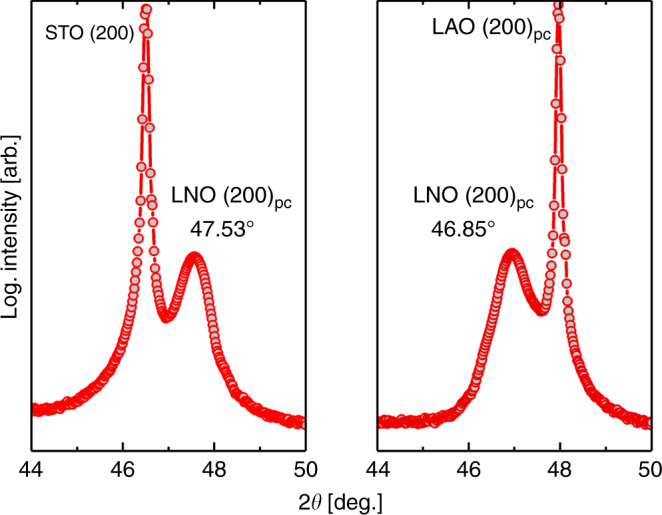
(200)_pc_ peak position of as-deposited LaNiO_3_ on SrTiO_3_ (100) and LaAlO_3_ (100) substrates. X-ray diffractogram of the LNO (200)_pseudocubic_ reflections for **a** SrTiO_3_(100)||LaNiO_3_(100)_pc_ and **b** LaAlO_3_(100)_pc_| |LaNiO_3_(100)_pc_ accompanied by the (200) substrate reflections for SrTiO_3_ and LaAlO_3_ (marked by *). Actual datapoints are marked by filled circles, and connected with straight lines. Data collected on 30-nm-thick films.

The direction of strain can be confirmed by studying the out-of-plane lattice parameter of LNO in the two substrate systems. Assuming a (near) isovolumetric strain process, tensile in-plane strain leads to compression of the *c*-axis (out-of-plane), whereas compressive in-plane strain leads to expansion of the *c*-axis. This is what is observed for the STO(100)||LNO(100)_pc_ and LAO(100)_pc_||LNO(100)_pc_ systems (Fig. 2). On STO (100) the film exhibits an out-of-plane lattice parameter of 3.822 Å (0.5% compressive strain), while on LAO (100)_pc_ the same parameter is 3.875 Å (0.9% tensile strain). In other words, the *c*-axis is more strained on LAO (100)_pc_ than on STO (100).

We carried out a Williamson−Hall (WH) analysis of the broadening for three specular reflections to deconvolute broadening from out-of-plane strain and crystallite size. The out-of-plane crystallite size from the WH-analysis was estimated to be 38 nm on LAO (100)_pc_ and 32 nm on STO (100), with a slightly larger *c*-axis strain on LAO (100)_pc_. This is consistent with the direct measurement of the *c*-axis by XRD. The crystallite size is WH-estimated to be close to the thin film thickness, pointing towards continuous crystals traversing all the way from the substrate to the film surface.

Specular XRD of select samples on SrTiO_3_ (100) annealed at 650 °C in air was carried out to investigate any relaxation of the imposed strain that was observed for the as-deposited samples (Supplementary Fig. 5). While the thin film *c*-axis was slightly compressed upon annealing (Δ2*θ* ≈ +0.2°, Δ*c* ≈ −0.013 Å), very little additional crystallization was observed. Some additional fractional fringes are observed, indicative of a somewhat sharper interface and/or surface. Tuning the strain of the as-deposited epitaxial films by post-deposition annealing is an intriguing possibility, but one that is left for later investigation.

**Fig. 3:**
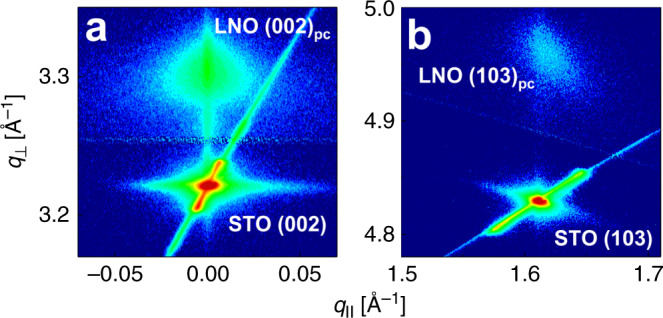
Reciprocal space maps of as-deposited LaNiO_3_ on SrTiO_3_ (100) substrates. Reciprocal space maps of **a** the specular LaNiO_3_ (002)_pc_ on SrTiO_3_ (001) and **b** the asymmetric LNO (103)_pc_ on SrTiO_3_ (100). The color scheme is on a linear scale. Data collected on 30-nm-thick films.

We further carried out RSM of a specular (002) and an asymmetric reflection (103) to determine the epitaxial relationship (Fig. 3). The position of the reflections reveals the expected cube on cube orientation, with a stringent in-plane coordination (e.g. STO(100)|STO[100]||LNO (100)_pc_|LNO[100]_pc_). Also note that the film scatters at the same *q*_||_ as the substrate, showing that the film in-plane lattice parameters are strained to be equal to those of the substrate, as expected by the epitaxial relation. We finally carried out a *φ*-scan of the LNO (103)_pc_ reflection to investigate the in-plane correlation between the substrate and the film (Supplementary Fig. 6). The (103)_pc_ reflection is clearly four-fold and coupled to the substrate, once again confirming the stringent epitaxial relationship.

### Structural analysis by scanning transmission electron microscopy

**Fig. 4:**
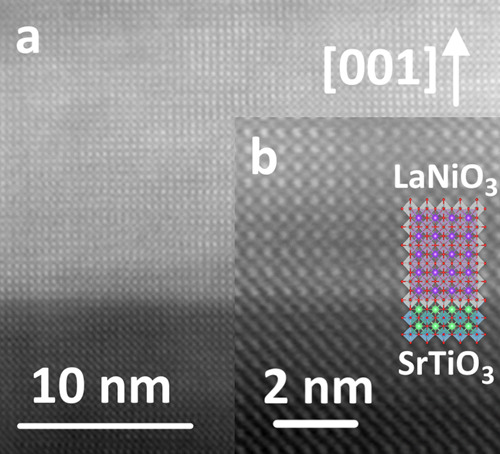
Scanning transmission electron micrograph of as-deposited LaNiO_3_ on SrTiO_3_ substrates. HAADF-STEM image of the LNO thin film on STO. **a** shows a large defect-free area. **b** shows a magnified local environment with the LNO and STO unit cells overlay to emphasize the extremely sharp interface that is observed.

The determination of macroscopic epitaxy as observed by a variety of XRD-methods calls for a local investigation by transmission electron microscopy (TEM). High-angle annular dark field scanning TEM (HAADF-STEM) micrographs of the interface between STO and LNO show the local epitaxial quality (Fig. 4). These micrographs show that the interface is sharp, with well-defined epitaxy, and that there are very few structural defects. On low magnification, a few voids (approx. Ø 10 nm) can be observed in the film (Supplementary Fig. 7). The origin of these voids is not clear; however, this is quite commonly observed in thin films made by gaseous deposition and likely stem from encapsulation of either purging gas or byproducts during deposition^37^. They do not seem to affect the functional properties of the films.

**Fig. 5:**
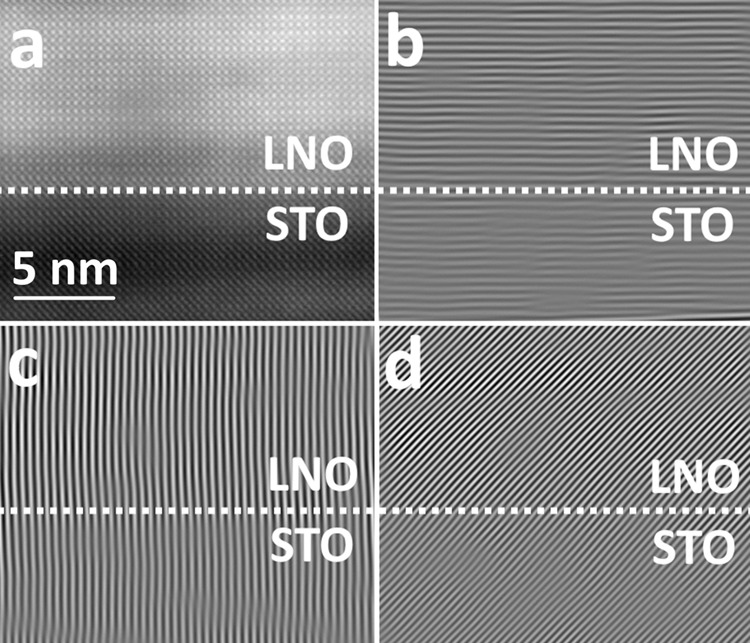
Fourier filter analysis of scanning transmission electron micrographs. **a** HAADF-STEM image of the LNO||STO interface, showing the region in which the Fourier analysis was carried out. **b** Horizontally Fourier-filtered. **c** Vertically Fourier-filtered. **d** Diagonally Fourier-filtered.

The existence of defects was evaluated by carrying out a direct Fourier transform and ensuing analysis in vertical, horizontal and diagonal directions, showing that the film is near defect free over large areas (Fig. 5). This means that the film has continuously grown epitaxially throughout the deposition, since we did not apply any post-deposition annealing that could have facilitated solid phase epitaxy. Furthermore, solid phase epitaxy on as-deposited amorphous films usually result in interface- and surface roughening as well as introduction of structural defects, which is often detrimental to the sharp interfaces that are needed in a device. The STEM analysis shows that our films are flat and exhibit sharp interfaces, which is essential in multilayer device engineering.

### Chemical analysis by X-ray photoelectron spectroscopy

**Fig. 6:**
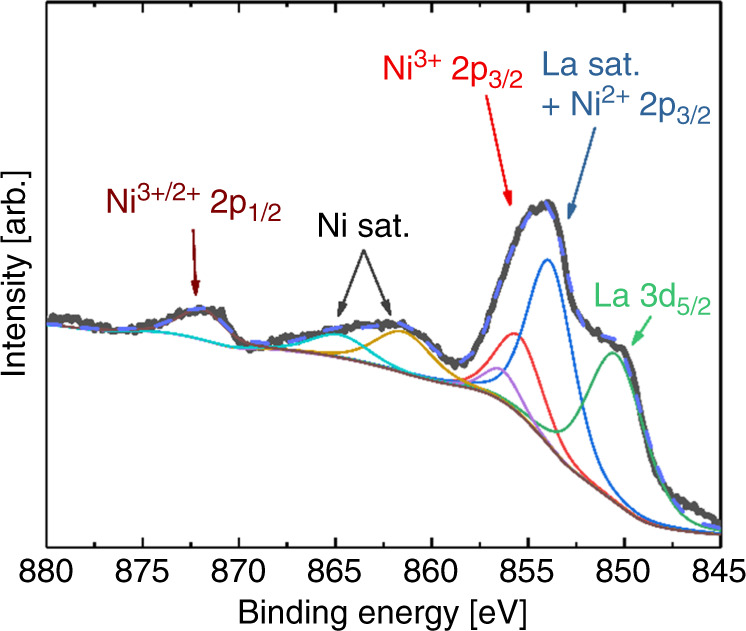
X-ray photoelectron spectroscopy of Ni 2p in LaNiO_3_ on SrTiO_3_ substrates. XPS of Ni 2p (intertwined with La 3d_5/2_) showing a complex pattern of intensities from Ni satellites, Ni^2+/3+^ 2p_3/2_ and 2p_5/2_, La 3d_5/2_ and La satellites. The dashed blue line is the total fit.

We carried out an X-ray photoelectron spectroscopy (XPS) analysis for films deposited on SrTiO_3_ (100) of Ni 2p (intertwined with La 3d) in an attempt to study the Ni^3+^ to Ni^2+^ ratio (Fig. 6). In stoichiometric LNO the nominal oxidation state of Ni is 3+, but there will always be some reduction of the surface forming an oxygen-deficient LaNiO_3−*δ*_ phase. Unfortunately, the overlap between satellite peaks from La 3d make a quantitative analysis difficult. However, using prior literature on close to perfect bulk LNO, we were able to make a comparison pointing towards a high amount of Ni^3+^, with a composition of at least LaNiO_2.8_^38^. Note that this is the composition in the top nanometers of the film, and that it is very probable that there is an even higher concentration of Ni^3+^ towards the substrate interface.

We further used a peak fitting procedure on a high-quality survey spectra to confirm the composition of the films (Supplementary Fig. 8). This analysis points towards a composition of La_1.01_Ni_0.99_O_2.95_, which again supports a high amount of Ni^3+^ in the films. It should also be mentioned that the oxygen content here is likely to be slightly overestimated due to the existence of carbonate species on the surface, as there is a small fraction of carbon (<2 at.%) as analyzed by the survey spectrum.

### Electrical properties

With confirmation that the structural and chemical quality of the LNO films are high, we turned towards an investigation of the electrical properties. Four-point probe measurements (point distance 1 mm) in room temperature reveal metallic electrical resistivity. STO(100)||LNO(100)_pc_ exhibits a resistivity of ~100 µΩ cm on average, with a lowest measured resistivity of 80 µΩ cm. This is below the reported values for bulk LNO, which is explained by the tensile strain that LNO is perturbed by an STO, increasing the Ni−O−Ni bond angle and widening the bands. Note that post-deposition annealing of these samples provides very little added functionality, with lowest measured resistivity of 70 μΩ cm. For LAO(100)_pc_||LNO(100)_pc_ the average resistivity measured was ~300 µΩ cm, which is slightly higher than bulk LNO. This is explained by the compressive strain, decreasing the Ni−O−Ni bond angle and narrowing the bands. Note that films down to a thickness of 2.5 nm has been characterized and found to conduct with similar resistivity as the 30 nm films that are presented here.

**Fig. 7:**
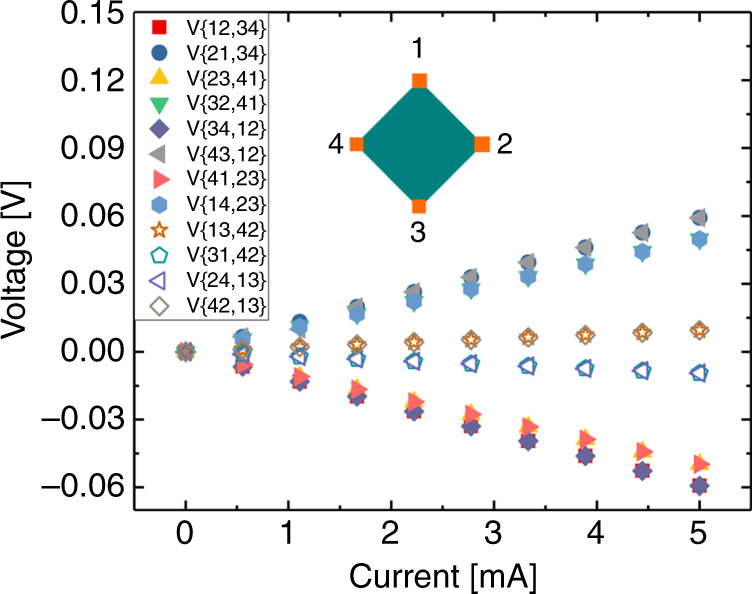
Hall measurements for deduction of carrier density. Voltage vs. current in 12 configurations of source and drain. Legends are according to the numbering shown in the measurement setup inset. Hard symbols represent the standard ohmic measurements used to deduce film resistivity. The open symbols represent the induced Hall voltage used to deduce carrier density.

To further investigate the electrical properties of the sample, we carried out a Hall analysis using a four-point setup where each corner of the 1 ∙ 1 cm^2^ sample is contacted to a probe (Fig. 7). The film is perturbed by a 1.02 T magnet, which allows for measurements of induced Hall currents, and deduction of carrier densities.

The resistivity was estimated to be 138 µΩ cm for STO (100)||LNO(100)_pc_ at ambient, which is slightly higher than measured by the four-point probe. This is likely because the Hall-setup uses a much larger probe distance (10× longer), which will be less tolerant to any grain boundaries that may exist in the film. It should still be noted that the resistivity is on par with that of bulk LNO (~120 µΩ cm)^15^.

The induced Hall voltage is measured to be ~ 2 mV A^−1^. Using the film conductivity, sample thickness, current and magnetic field, a Hall coefficient of 0.0549 mm^3^ C^−1^ and a charge carrier density of ~3.6 · 10^22^ cm^−3^ can be deduced. This is very close to the theoretical 3.4 · 10^22^ cm^−3^ carriers that would be present if each NiO_6_-octahedron contributes with one carrier each. High carrier density is key for materials that are to be used as gates in oxide electronics.

**Fig. 8:**
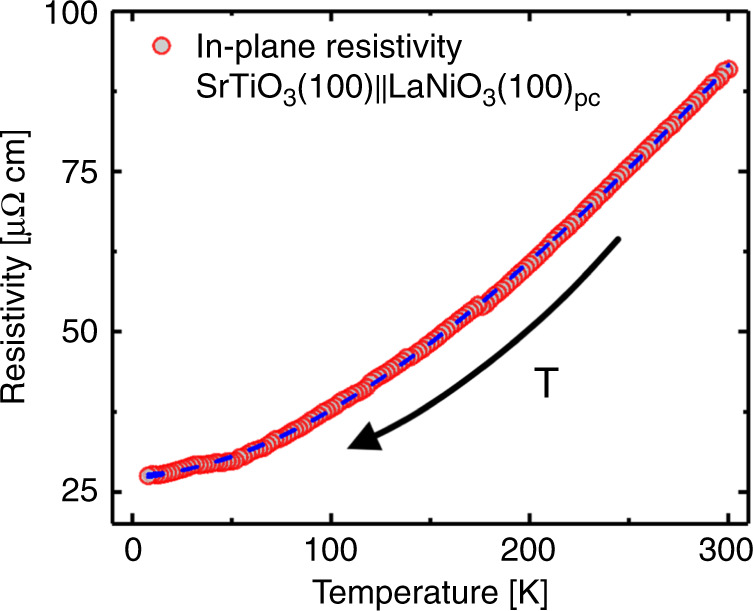
Temperature-dependent in-plane resistivity. Temperature dependence of the SrTiO_3_(100)||LaNiO_3_(100)_pc_ film in-plane resistivity as measured by four-point PPMS measurements. The temperature was swept from RT to 6 K by means of liquid He, and the resistivity was measured for every 2 K. The small bump around 50 and 180 K is likely a result of deteriorating surface adhesion of the sputtered gold contact. The blue dashed line corresponds to a linear combination of polynomials based on Matthiessen’s rule, given by the polynomial $\rho \left( T \right) = 27.6 + 0.012T^2 + 2.02 \cdot 10^{ - 5}T^3 + 4.09 \cdot 10^{ - 12}T^5$.

We measured the temperature-dependent resistivity of the LNO films on STO using a PPMS cooled by liquid He. The temperature was swept from RT to 6 K and the resistivity was collected for every 2 K (Fig. 8).

The decrease in resistivity as a function of temperature underpins that the films are metallic across the whole temperature range. LNO with significant oxygen under-stoichiometry will exhibit an MIT at a set temperature. Thus, the temperature-dependent resistivity complements our previous identification that our method can be used to deposit films with both high chemical and structural quality (i.e. low oxygen under-stoichiometry). Note that resistivity measurements were carried out for pristine films and films that have been exposed to humidity over a long time, with no apparent degradation of the functionality.

Finally, we fitted the temperature dependence of the resistivity to a linear combination of fifth-, third- and second-order polynomials to identify the mechanism for resistivity, in compliance with Matthiessen’s rule. The dependence is strongly correlated to the second-order polynomial ($\rho = 27.6 + 0.012T^2 + 2.02 \cdot 10^{ - 5}T^3 + 4.09 \cdot 10^{ - 12}T^5$ µΩ cm), indicating that the resistivity is mainly governed by electron−electron interactions. This is as expected in a correlated electron system like LaNiO_3_. The constant term, $\rho _0$, is found to be 27.6 µΩ cm, which is slightly higher than previously reported for single crystals. This may be a result of the previously described oxygen vacancies, which has a higher concentration towards the film surface^15^.

## Discussion

We have developed an ALD process for LaNiO_3_ that can be used to obtain highly epitaxial thin films on LaAlO_3_ and SrTiO_3_ substrates. The films are epitaxial as deposited at 225 °C and need no further post-deposition annealing to obtain the highly regarded functional properties. The films are nearly defect free as shown by XRD, X-ray photoelectron spectroscopy and TEM. They conduct electrons with low resistivity (~10^−4^ Ω cm) and with high charge carrier density (~3 · 10^22^ cm^−3^). The imposed strain from the substrate affects the electrical properties by changing the in-plane Ni−O−Ni bond angle, and thus the width of orbital bands.

**Fig. 9:**
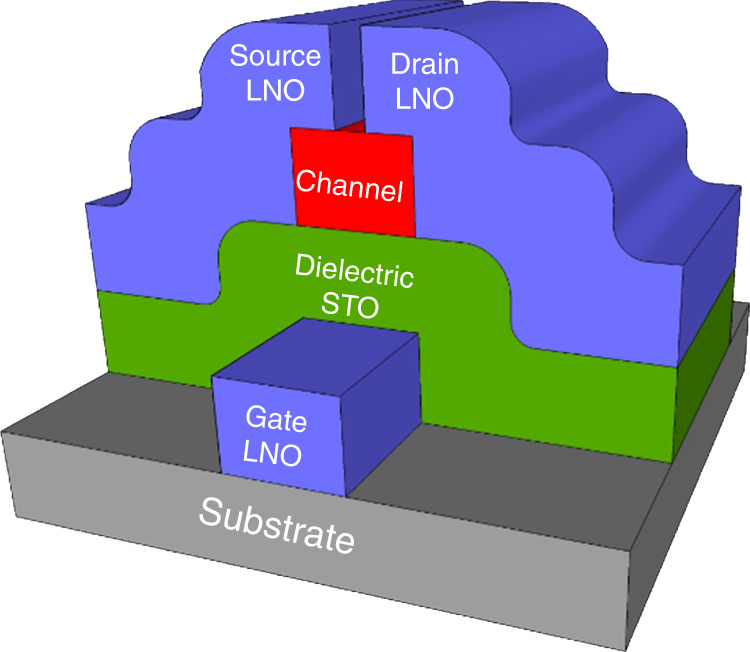
Example architecture of complex oxide field effect transistor. Example of architecture for a layered all-oxide COFET using metallic LaNiO_3_ as source, drain and gate, and SrTiO_3_ as the gate oxide. The choice of channel material is still an open question and will depend on the device, but recent literature suggests e.g. SmNiO_3_ for multilevel memristive devices^39–41^.

The applicability of LaNiO_3_ thin films in device construction has been significantly hampered by the need for high deposition temperatures or high-temperature post-deposition annealing. This new process maintains a low thermal budget that should be compatible with most device fabrication, and especially in monolithic device integration in transistor technology. The direct low-temperature deposition of LaNiO_3_ on SrTiO_3_ at 225 °C marks a fundamental step in realizing SrTiO_3_ gate oxides, and furthermore as an electrode in future all-oxide electronics, e.g. in COFETs (Fig. 9).

## Methods

### Atomic layer deposition

Thin film depositions were carried out in an F-120 Sat ALD reactor (ASM Microchemistry). The deposition temperature was 225 °C with an operating pressure of 2.4 mbar, maintained by a 300 cm^3^ min^−1^ primary flow rate of N_2_. Nitrogen was supplied from gas cylinders (Praxair, 99.999%) and run through a Mykrolis purifier for removal of any oxygen or water contamination.

La(thd)_3_ (Volatech, 99%) and Ni(acac)_2_ (Sigma Aldrich, 97%) were used as cation precursors (acac = acetylacetonate). Both precursors were supplied from open boats inside the reactor, and maintained at 185 °C throughout the deposition. Ni(acac)_2_ was resublimated at 175 °C for purification prior to use in the reactor. The cation precursors were pulsed into the reaction chamber by means of inert gas valves. O_3_ was used as the oxygen source, made from O_2_ (Praxair, 99.5%) using an AC-2505 (In USA) ozone generator supplying 15 wt.% O_3_ in O_2_. Pulse durations were 2, 2 and 4 s for La(thd)_3_, Ni(acac)_2_ and O_3_, respectively. Purge durations were 2 s after cation precursor pulses, and 3 s after the ozone pulses. Confirmation of self-limiting behavior for the employed pulse- and purge scheme is shown in Supplementary Fig. 2.

The thin films were deposited on 1 · 1 cm^2^ Si for routine characterization of thickness and 3 · 3 cm^2^ Si for analysis of conformality and cation stoichiometry. Selected compositions were deposited on LaAlO_3_ (LAO) (100) (Crystal GmbH), (110) and (111) (MTI Corp.) and SrTiO_3_ (STO) (100) (Crystal GmbH), (110) and (111) (MTI Corp.) single crystals for facilitation of epitaxial growth.

### Thickness analysis

Routine measurements of thickness were carried out on an alpha-SE spectroscopic ellipsometer (J.A. Woollam) in the 390–900 nm wavelength range. We successfully employed a Cauchy function to model the collected data. X-ray reflectivity (XRR) was used as a complementary method to determine thickness for very thin films (<10 nm). XRR was carried out on an Empyrean diffractometer (Panalytical) equipped with a Cu Kα_1_ (*λ* = 1.5406 Å) source powered at 45 kV and 40 mA, and a parallel beam mirror.

### Chemical and compositional analysis

Chemical composition was analyzed using an Axios Max Minerals X-ray fluorescence (XRF) system (Panalytical), equipped with a 4 kW Rh-tube. The system runs using Omnian and Stratos options for standardless measurement of thin films. Complementary compositional analysis and evaluation of chemical environment was carried out by XPS, using a Theta Probe Angle-Resolved XPS (Thermo Scientific). This instrument is equipped with an Al Kα (*hν* = 1486.6 eV) source and the analysis chamber was maintained at ~10^−8^ mbar during analysis. Pass energy values of 200 and 60 eV were employed for survey and detailed scans, respectively. The spectra were analyzed using the Thermo Avantage software suite.

### X-ray diffraction

X-ray diffraction for structural analysis was carried out on an AXS D8 Discover (Bruker) diffractometer, equipped with a LynxEye strip detector and a Ge (111) focusing monochromator, providing CuKα_1_ (*λ* = 1.5406 Å) radiation. Recriprocal space maps were collected with a Pixcel 3D detector on an Empyrean diffractometer (Panalytical) equipped with a Cu Kα_1_ (*λ* = 1.5406 Å) source powered at 45 kV and 40 mA, and a primary Barthels monochromator.

### Scanning transmission electron microscopy

Scanning transmission electron microscopy was carried out using a JEOL 2100F microscope at 200 kV. Sample preparation was carried out by mechanical polishing and ion milling on a Gatan PIPS II. Collected high-angle annular dark field (HAADF) images were treated and Fourier-filtered using the Gatan Microscopy Suite software.

### Electrical characterization

Room-temperature resistivity measurements were carried out using a four-point probe and a Keithley model 2400 SourceMeter. The sheet resistivity was recorded by measuring resistance in ten points from 1 to 10 µA. Variable temperature resistivity measurements were performed on a Model 4000 physical property measurement system (PPMS, Quantum Design). The samples were mounted on a puck and contacted with gold wires on gold pads deposited by evaporation. Resistivity was collected in a four-point setup, while the temperature was swept from 300 to 6 K.

## Supplementary information

Supplementary Information

Peer Review File

## Data Availability

The data sets generated and analyzed during the current study are available from the corresponding author upon reasonable request.
