# Supplementary material for: A foundation for complex oxide electronics -low temperature perovskite epitaxy
Source: Nat Commun. 2020 Jun 8;11:2872. doi: 10.1038/s41467-020-16654-2 (PMC7280286; doi:10.1038/s41467-020-16654-2)
Supplement: Supplementary file 1 — Supplementary Information [file 41467_2020_16654_MOESM1_ESM.pdf]

Supplementary Information

A Foundation for Complex Oxide Electronics: Low Temperature Perovskite Epitaxy

Henrik. H. Sønsteby[1]*, Erik Skaar[1], Øystein S. Fjellvåg[2], Jon E. Bratvold[1], Helmer Fjellvåg[1] and Ola Nilsen[1]

[1]Department of Chemistry, Center for Materials Science and Nanotechnology, University of Oslo, Blindern, N-0315 Oslo, Norway

[2]Department for Neutron Materials Characterization, Institute for Energy Technology, N-2007 Kjeller, Norway

*Corresponding e-mail: henrik.sonsteby@kjemi.uio.no

Supplementary Figure 1: Optimizing the Process for Stoichiometry

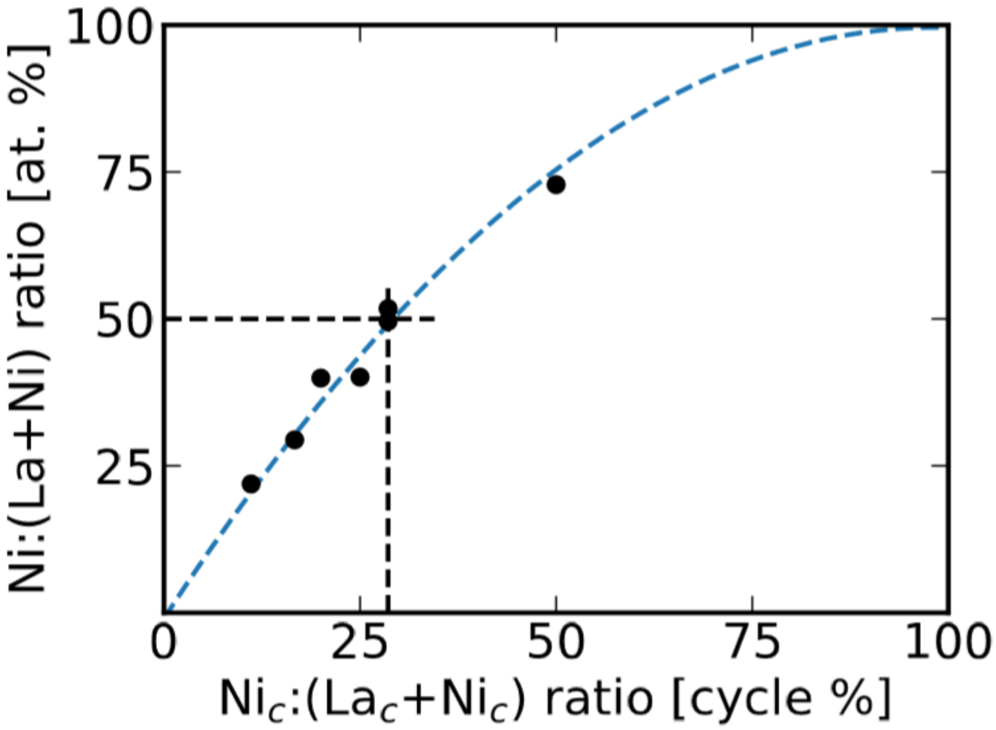

Supplementary Figure 1: Correlation between pulsed cation precursor ratios ($Ni(acac)_2/(Ni(acac)_2+La(thd)_3)$) and deposited cation ratios Ni/(Ni+La) as measured by x-ray fluorescence spectrometry. The blue line is a second order polynomial fit.

Supplementary Figure 2: Self-limiting Behaviour

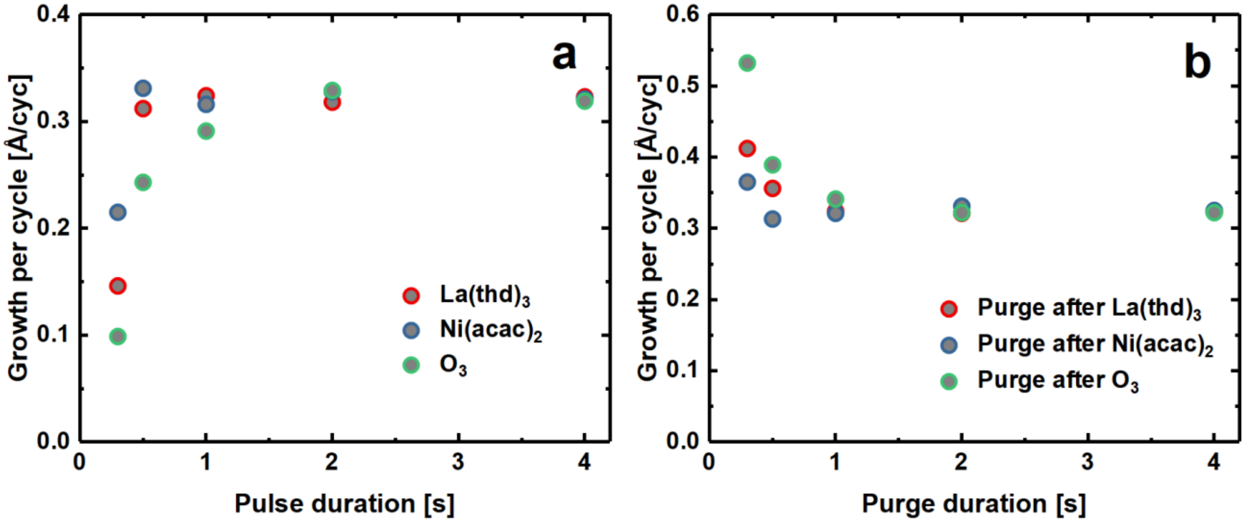

Supplementary Figure 2: Effects of pulse- and purge duration within the complex process for the employed precursors when all other time parameters are kept excessively long (4 s for metals, 6 s for O_3). This data was collected for a 1:1 pulsing scheme.

Supplementary Figure 3: Amorphous $LaNiO_3$ on Si (100) substrates

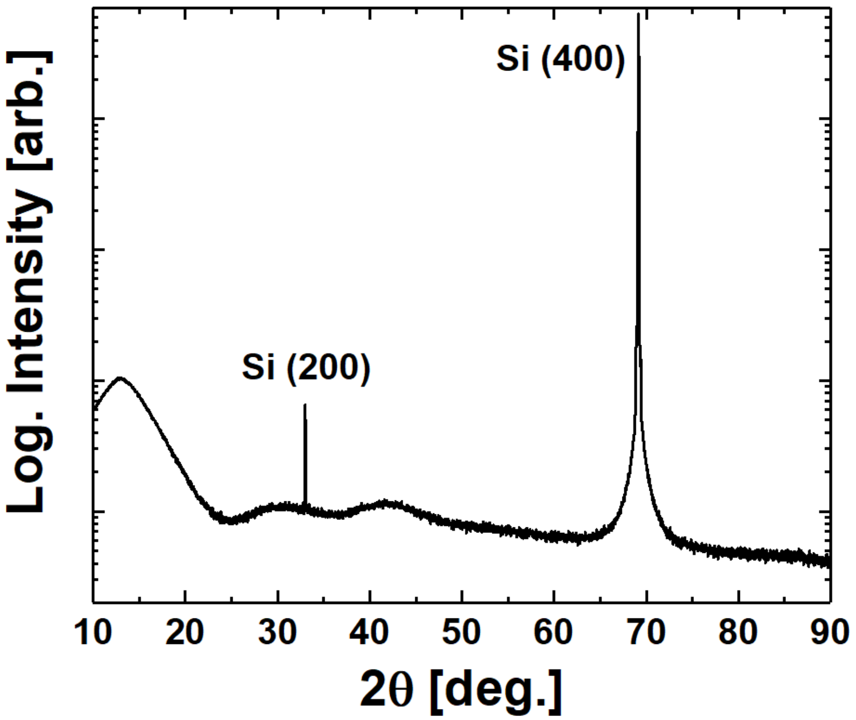

Supplementary Figure 3: X-ray diffractogram of as deposited (225 °C) $LaNiO_3$ thin films on SiO_x||Si (100) substrates, showing no Bragg reflections from the films.

Supplementary Figure 4: Oriented $LaNiO_3$ on a Range of Substrate Orientations

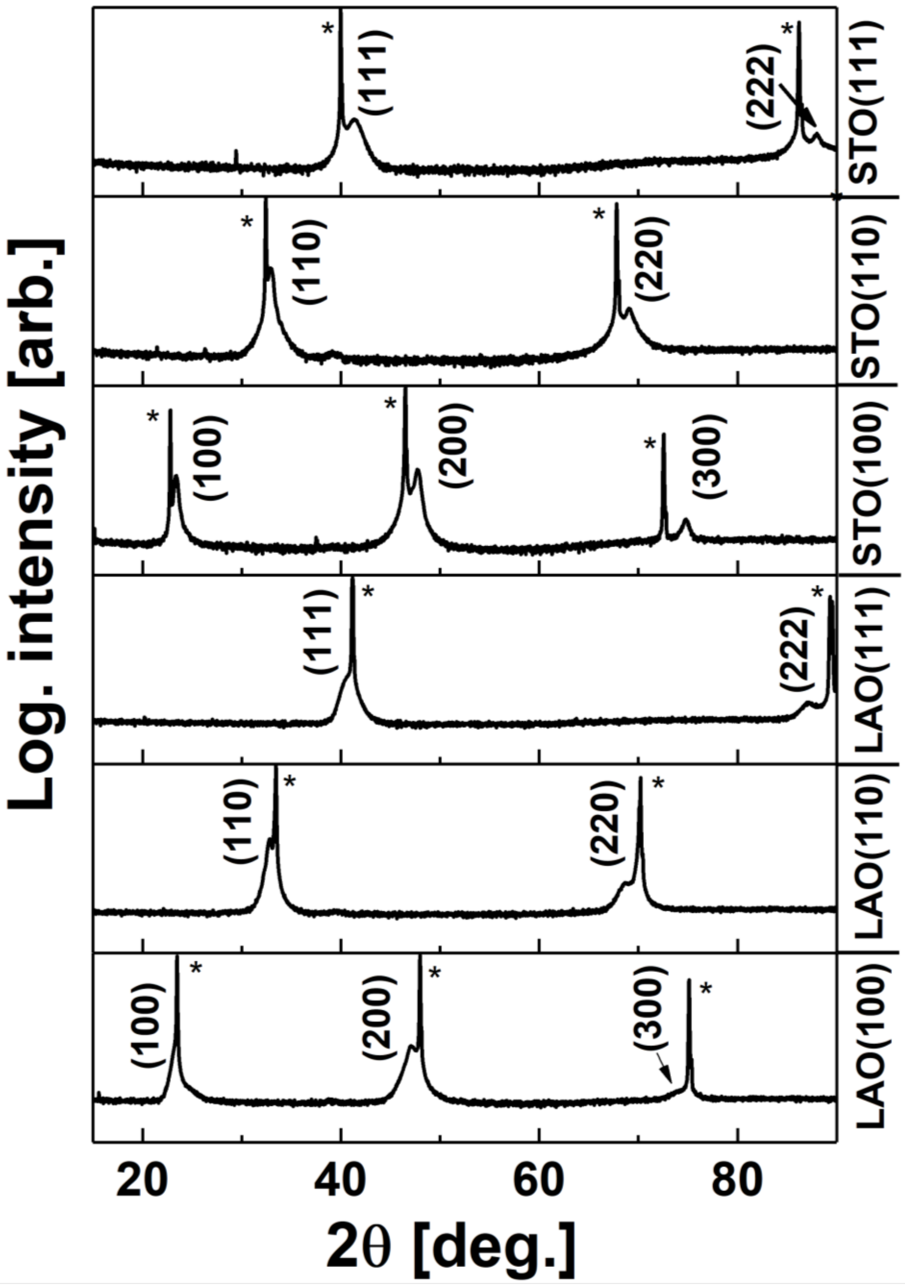

Supplementary Figure 4: X-ray diffractograms of as deposited (225 °C) $LaNiO_3$ thin films on $LaAlO_3$ (100), (110) and (111) and $SrTiO_3$ (100), (110) and (111) substrates, showing bragg reflections from single oriented $LaNiO_3$. Substrate reflections are marked by *.

Supplementary Figure 5: Effect of Annealing on Crystallinity of $LaNiO_3$ on $SrTiO_3$ (100)

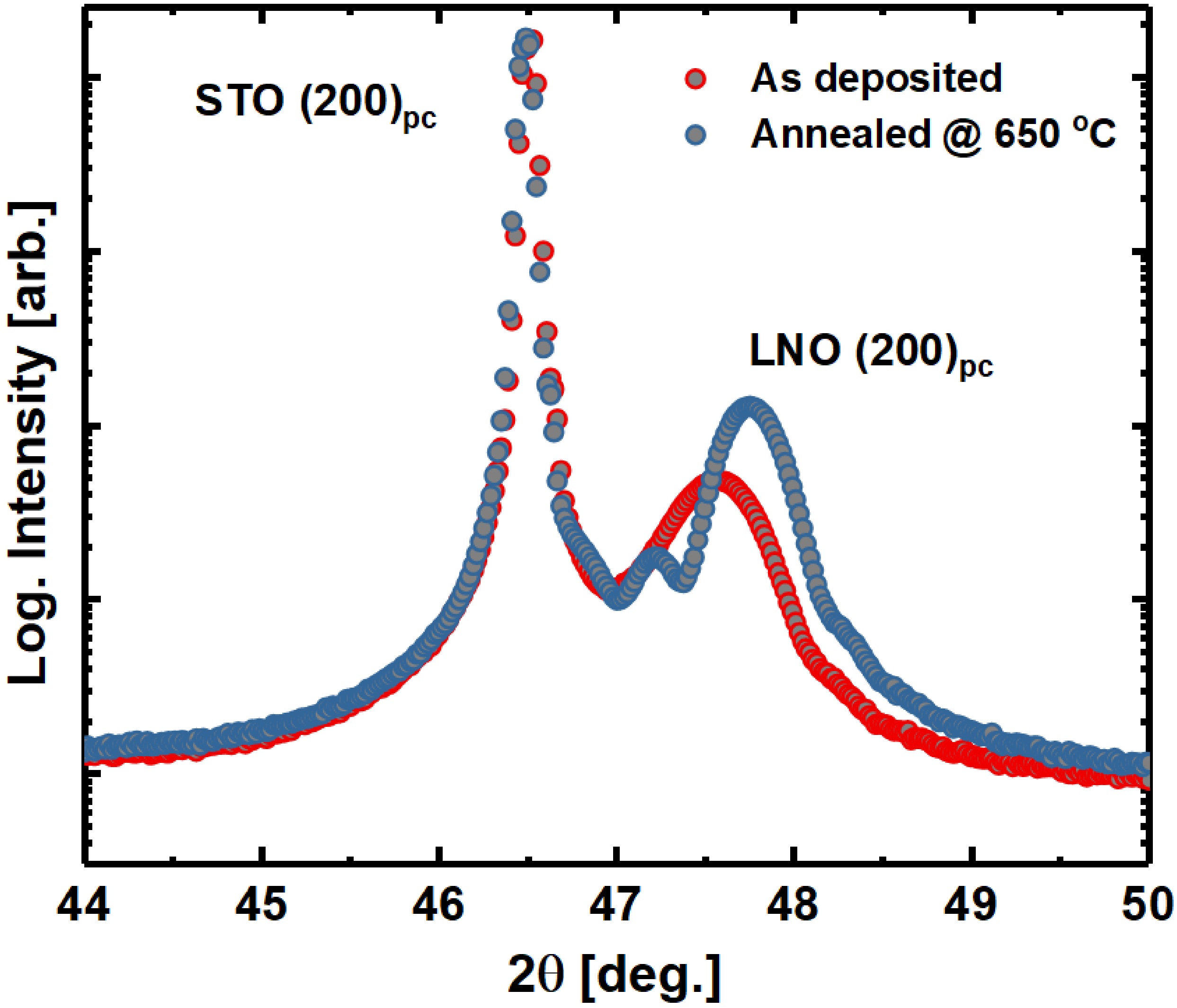

Supplementary Figure 5: Specular XRD of the $LaNiO_3$ (200) reflection for thin films on $SrTiO_3$ (100) substrates for as deposited (red) and 650 °C in air post deposition annealed (blue) samples.

Supplementary Figure 6: φ-scan of Epitaxial $LaNiO_3$ $(100)_{pc}$||$SrTiO_3$ (100)

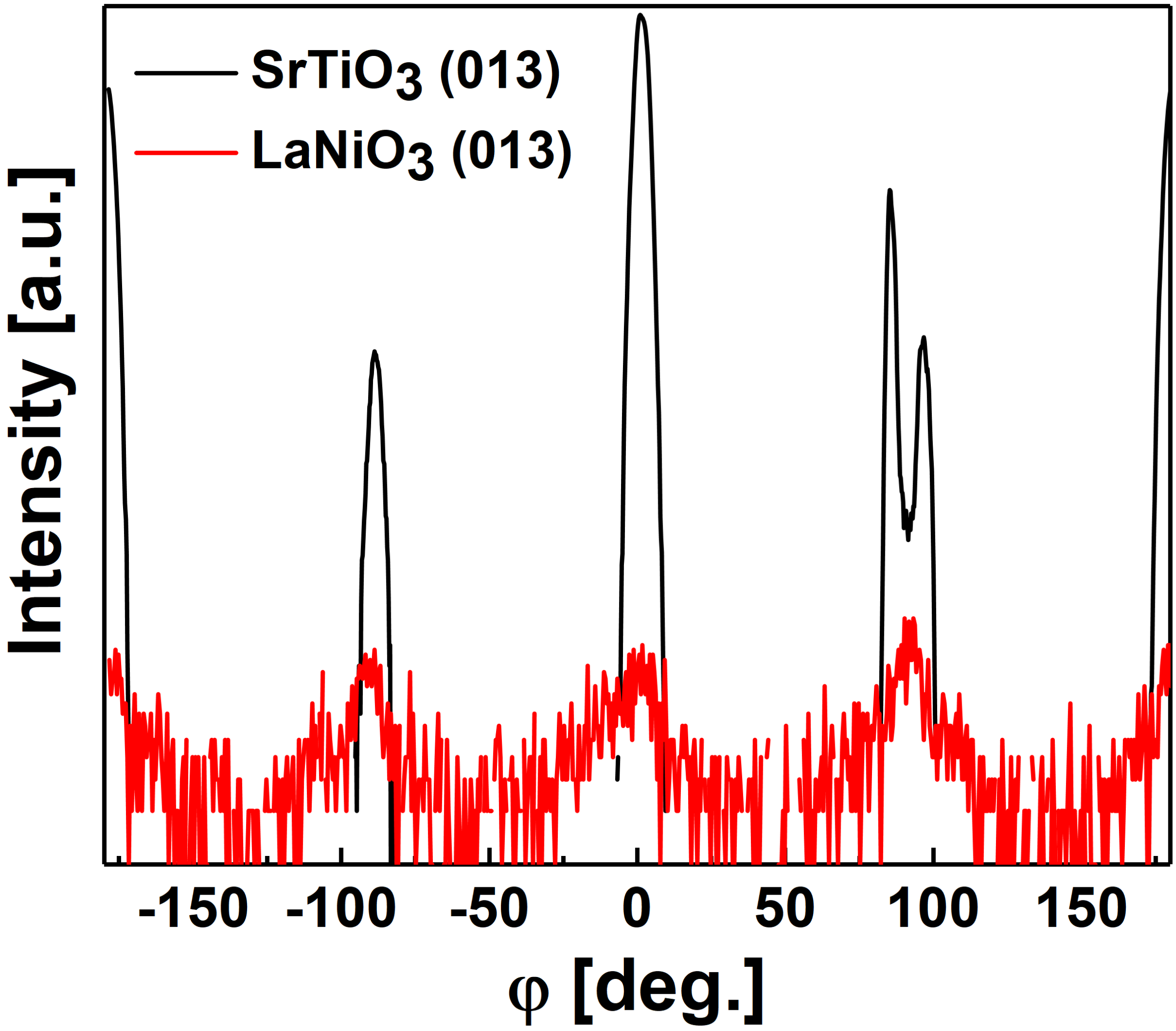

Supplementary Figure 6: ϕ-scan of the (013) family of reflections for as deposited (225 °C) $LaNiO_3$ thin films on $SrTiO_3$ (100).

Supplementary Figure 7: Large Area Transmission Electron Micrograph

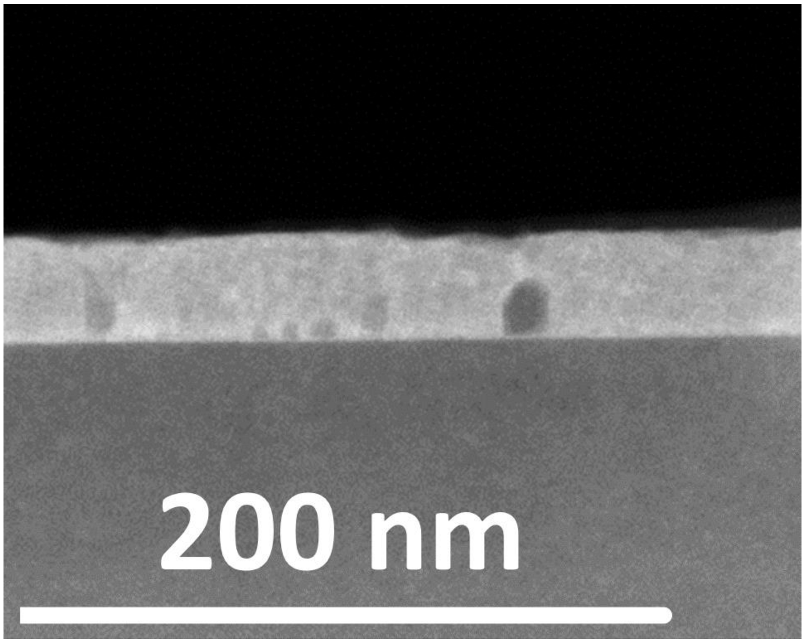

Supplementary Figure 7: Low-magnification HAADF-STEM image of 30 nm $LaNiO_3$ on $SrTiO_3$ (100), the same sample used for high-magnification HAADF-STEM imaging in figure 5 and 6 of the main manuscript. The interface is inherently sharp over large distances.

Supplementary Figure 8: X-ray Photoelectron Spectroscopy Survey Scan of $LaNiO_3$ (100) on $SrTiO_3$ (100)

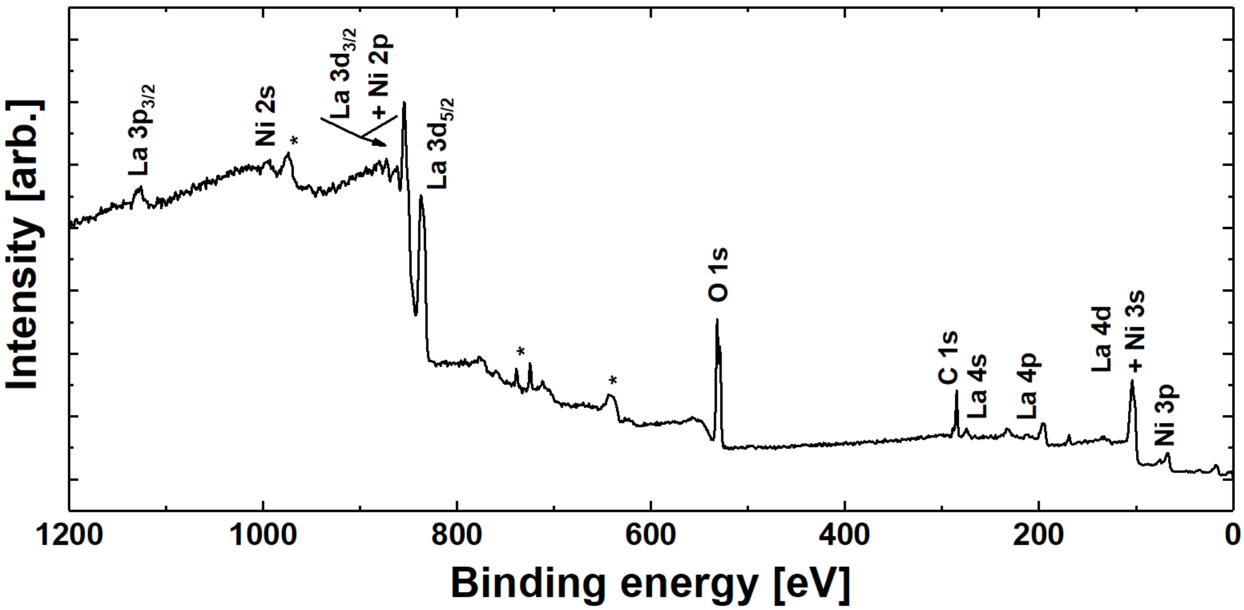

Supplementary Figure 8: X-ray photoelectron spectroscopy survey spectrum of $LaNiO_3$ thin films on $SrTiO_3$ (100) substrates.
