# Supplementary material for: A foundation for complex oxide electronics -low temperature perovskite epitaxy
Source: Nat Commun. 2020 Jun 8;11:2872. doi: 10.1038/s41467-020-16654-2 (PMC7280286; doi:10.1038/s41467-020-16654-2)
Supplement: Supplementary file 2 — Peer Review File [file 41467_2020_16654_MOESM2_ESM.pdf]

Reviewers' comments:

Reviewer #1 (Remarks to the Author):

The manuscript addresses the emerging area of all-oxide electronics and one of the major challenges in integration, namely the very limited availability of low temperature synthesis routes. The authors successfully select the case study of $LaNiO_3$ and demonstrate a low-temperature ALD route towards low temperature epitaxial growth of electrically conductive $LaNiO_3$ on $SrTiO_3$ and $LaAlO_3$.

The manuscript is extremely well written and it leads to original and well convincing results.

The following improvements are suggested here for the final version of the manuscript:
- The authors select for their study the O_3 route. An alternative could have been the O_2 plasma route, also valid when processing at low T is required. Moreover, the selection of the La- and Ni-precursors is not motivated. Other precursors, for example with Cp ligands in the case of Ni, are available too. Could the authors comment on their selection of the metal precursors and co-reactant choice, in this respect?

-In the Methods section the authors introduce the ALD recipe in terms of pulse and purge scheme as based on previous studies with the same precursors. It would be extremely interesting to add details on this matter: is self-limiting growth guaranteed also within the super-cycle towards $LaNiO_3$?

- The stoichiometry analysis is primarily carried out by XRF and XPS. The latter is characterized by a complex spectrum as shown in Fig.5. Would RBS analysis shed more light and accuracy on the determination of the stoichiometry?

- Given the hygroscopic character of La_2O_3, did the authors experience any instability in film properties for $LaNiO_3$?

Reviewer #2 (Remarks to the Author):

The authors report the use of ALD to obtain epitaxial growth of $LaNiO_3$ at low temperature (225ºC) with competitive physical properties. This is a bourgeoning area of research and these results will certainly contribute to speed up the integration of complex oxides on silicon technology.
Here below I point several comments and suggestions to improve the manuscript

1) Page 2 line 45: the authors mention that in ABO_3, A = small metal cation, B= large metal cation. This is not a general rule, in fact, for the case study (LNO) the ionic radius of La >> ionic radius of Ni. Many other examples can be mentioned here where A cation is much larger than B cation : $BiFeO_3$, $BaTiO_3$...

2) What is the role of the precursor chemistry on favoring epitaxial growth? why the use of $Ni(acac)_2$ promotes epitaxy over $Ni(thd)_2$? Would the use of $La(acac)_3$ further improve the growth ? The authors should comment on how this effect the growth mechanism

3) Did the authors try to post anneal the samples and study if they are still strained? It could be good to know if this post thermal treatment changes the structural defect scenario and thus the resistivity behavior.

4) It is reported that at very thin films (ultrathin), some materials show a “dead-layer” and they are insulating. Did the authors study which is the minimum thickness by which the samples start to be conductive? That is interesting to know to use the minimum material in monolithic device integration

5) Clearly ALD is a technique to prepare thin films and 30 nm is an “appropriate” thickness but, up to which thickness these ALD films are strained?

Reviewer #3 (Remarks to the Author):

I have carefully read the manuscript by Dr. Sønsteby and colleagues, and I believe it belongs in a more specialized journal.

The authors report a low temperature ALD growth of $LaNiO_3$ on $SrTiO_3$ and $LaAlO_3$ substrates. By itself, the ALD growth of perovskites is not particularly new. In particularly, LNO growth on STO has been recently reported by Ritala et al. What sets this work apart is crystallization upon deposition at 225C that eliminates the need for crystallization anneal (typically about 700C). And one could argue that this choice of precursors and oxidizer, resulting in low temperature process, makes it compatible with the back of the line (BOL) processing in Si CMOS. However, the authors are focusing on all oxide electronics, where this is not even an issue.

The paper is full of strong statements, such as that “oxide electronics is rising fast as a contender to Si-based circuitry,” or that the integration of LNO on STO is “a highly waited example in technology industry.” At best, these are artistic exaggerations.

To me, the paper shows low temperature crystallization (it does occasionally happen in ALD, for example $BaTiO_3$ on STO or even on Ge (001)) of LNO on STO and LAO. The films are partially relaxed, and show expected electrical behavior. The crystal quality is similar to that of a typical ALD film (plus some porosity). This is another process in the tool box of oxide electronics that has been hyped since mid-nineties and is yet to show some technological promise.

I do not recommend publication in Nature Communications.

NCOMMS-20-02259 – Response to Reviewers Comments

The authors would first of all like to thank all three reviewers for taking the time to evaluate the manuscript and are happy that the work was generally well received and considered worthy of publication after some revision. We understand that reviewers 1 and 2 see the novelty and high impact of this study and are grateful that they find the manuscript well written. The reviewers have contributed interesting and appropriate comments and questions, which will be answered in detail below.

We believe that the manuscript is clearer and carries broader impact with the revisions that we have carried out. While we hope reviewer 3 agrees on the impact upon seeing the comments from the other referees, we hope that we have been able to present an even clearer description of the novelty of the work as it stands now.

Please note that the editor's requests have also been implemented in the revised manuscript. This also implies to the forms that we have been asked to fill out and submit.

We have highlighted any changes that have been made in the manuscript as response to the comments or questions. Comments by the referees are written in *italic*, and our response is written in a normal font. All changes in the manuscript are highlighted.

Reviewer #1

The manuscript addresses the emerging area of all-oxide electronics and one of the major challenges in integration, namely the very limited availability of low temperature synthesis routes. The authors successfully select the case study of $LaNiO_3$ *and demonstrate a low-temperature ALD route towards low temperature epitaxial growth of electrically conductive* $LaNiO_3$ *on* $SrTiO_3$ *and* $LaAlO_3$.

The manuscript is extremely well written and it leads to original and well convincing results.

We are happy that the referee was generally happy with the manuscript and consider it well written, and that she/he has identified the novelty and impact of the work. The following is a detailed response to the valuable comments and objections that referee #1 has raised.

***

The authors select for their study the O_3 route. An alternative could have been the O_2 plasma route, also valid when processing at low T is required. Moreover, the selection of the La- and Ni- precursors is not motivated. Other precursors, for example with Cp ligands in the case of Ni, are available too. Could the authors comment on their selection of the metal precursors and co-reactant choice, in this respect?

This is an interesting comment raised by the referee. As will be discussed below, we have identified that the driving force for the epitaxy lies in the metal precursors (mainly Ni(acac)$_2$), meaning that the oxidant may be of less relevance. Of course, we would have liked to employ a milder route (*e.g.* using H_2O or O_2) to widen the applicability, but for the metal precursors of choice this is not possible. The referee raises an interesting idea with respect to O_2 plasma. We do not have the possibility to employ O_2 plasma at this point in time, and thus we focus on thermally assisted ALD. We note that a recent report by Napari *et al*. shows growth of NiO from Ni(acac)$_2$ and O_2 plasma, so using plasma should in principle also be possible for complex oxide growth involving Ni. There are currently no reports using La(thd)$_3$ and O_2 plasma, for this process the co-reactant of choice has been O_3. Collectively, we believe that O_3 is the most straightforward choice. We absolutely do not disregard O_2 plasma as an alternative, and believe this would make for an interesting study given the chance to employ plasma in our reactor.

With respect to the choice of Ni-precursor, there is a strong motivation for the use of Ni(acac)$_2$. This precursor exhibits high hygroscopicity, and readily forms Ni(acac)$_2$(OH$_2$)$_2$ to create an octahedrally coordinated Ni-center. Ni^{2+} is not stable in a square planar coordination like it would have in Ni(acac)$_2$. Upon removal of water, Ni(acac)$_2$ readily forms a trimer, [Ni(acac)$_2$]$_3$ (figure below).

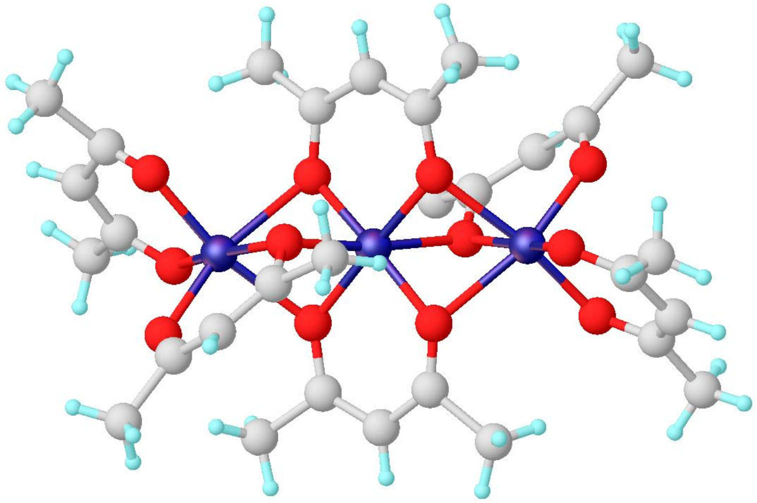

In this molecule, Ni-O-Ni bonds of similar length to what is found in $LaNiO_3$ is already present. We believe that this strongly affects the coordination in the deposited film and gives rise to the as deposited epitaxy that we observe. With water present, the trimer immediately decomposes to form the more stable $Ni(acac)_2(OH_2)_2$. Similar effects have not been observed for *e.g.* $Ni(cp)_2$ or $Ni(thd)_2$, hence the choice of $Ni(acac)_2$ as Ni-source.

To emphasize this effect in the manuscript, we have added the following:

Note that dry $Ni(acac)_2$ is known to sublimate in the form of an octahedrally coordinated trimer, $[Ni(acac)_2]_3$. In this trimer, Ni-O-Ni bonds of similar length to those found in LaNiO3 are already present, and this is believed to be of high importance with respect to epitaxial growth.

$La(thd)_3$ was chosen due to its successful use in previous deposition of *e.g.* $LaAlO_3$, $LaCuO_x$ and $LaMnO_3$.

We again thank the referee for this useful comment, and hope that our addition helps clarify and motivate the choice of precursor.

***

In the Methods section the authors introduce the ALD recipe in terms of pulse and purge scheme as based on previous studies with the same precursors. It would be extremely interesting to add details on this matter: is self-limiting growth guaranteed also within the super-cycle towards $LaNiO_3$?

This is of course highly relevant, and we are glad the referee does not just take our word for it. Self-limiting behavior is the watermark for ALD growth, and we have implemented pulse- and purge schemes that have been tested to exhibit self-limitation. Normally we would carry out such work using QCM, but the use of O_3 very often clouds the self-limitation. Thus, for this type of system we do old-school sets of depositions to find self-limiting process parameters.

In fact, our studies have shown that self-limitation is found (within complex growth), for pulse and purge durations that are much shorter than what we have employed. The chosen values are used for practical purposes, making absolutely sure that we do not get any gas phase reactions, and that the surfaces are saturated with precursors. While we do not think that ALD specifics is of high relevance to the paper as presented in Nature Comm., we understand that it is useful to have the data available.

To comply with this, we have added the following figures to the supplementary information:

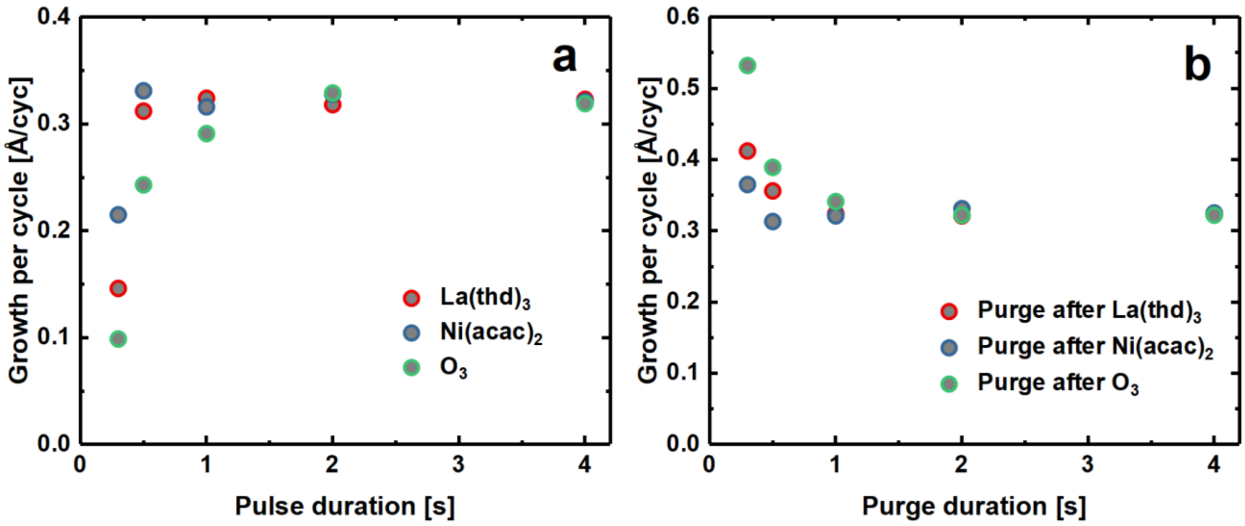

Figure B: Effects of pulse- and purge duration within the complex process for the employed precursors when all other time parameters are kept excessively long (4 s for metals, 6 s for O_3). This data was collected for a 1:1 pulsing scheme.

These data figures show the effect of pulse and purge duration within the complex process, when the all other time parameters are kept excessively long (4 s for metals, 6 s for O_3). This confirms the claimed self-limiting behavior, and also shows why we employ slightly longer pulse and purge times for O_3.

We have also removed the sentence about using the parameters due to previous studies, and added the following sentence to the manuscript methods part to comply with this change:

Confirmation of self-limiting behavior for the employed pulse- and purge scheme is shown in supporting information (Figure B).

We hope this clarifies our choice of pulse- and purge schemes.

***

The stoichiometry analysis is primarily carried out by XRF and XPS. The latter is characterized by a complex spectrum as shown in Fig.5. Would RBS analysis shed more light and accuracy on the determination of the stoichiometry?

This is an interesting comment by the referee, and one that we have previously considered since oxygen composition is always difficult to determine. Of course, the La:Ni ratio is properly defined by standardless XRF measurements, with very low uncertainty. The oxygen is the main challenge. XPS gives some quantitative information, but not with the same accuracy as for La:Ni from XRF. RBS could by all means be an alternative, but given the thickness of the films, the oxygen content would be clouded by oxygen signal from the substrates. Even with state-of-the-art RBS instrumentation, the depth resolution is somewhat limited, especially when the nominal oxygen content of the epitaxial films and the substrate is similar.

Furthermore, while the oxygen content is of course of interest, it is of minor importance as long as the $LaNiO_3$ films exhibit metallic resistivity at all temperatures. This simply means that the oxygen content is "high enough" to provide the wanted functionality.

No action was taken.

***

Given the hygroscopic character of La_2O_3, did the authors experience any instability in film properties for $LaNiO_3$?

This is another interesting question from the referee. The referee is right that La_2O_3 is of course very hygroscopic, and this is one of the challenges when employing it in ALD together with water-based processes. As an example, growth of $LaAlO_3$ using $La(thd)_3$ + O_3 and TMA + H_2O results in non-uniform growth and water reservoir effects. On the other hand, $LaNiO_3$ is not predominantly hygroscopic. *I.e.*, upon deposition of stoichiometric $LaNiO_3$, we do not find any evidence of change in functionality over long term exposure to ambient conditions and humidity.

We agree that this is of high relevance, and have added the following to the manuscript on page 13:

Note that resistivity measurements were carried out for pristine films and films that have been exposed to humidity over a long time, with no apparent degradation of the functionality.

***

Reviewer #2

The authors report the use of ALD to obtain epitaxial growth of LaNiO3 at low temperature (225ºC) with competitive physical properties. This is a bourgeoning area of research and these results will certainly contribute to speed up the integration of complex oxides on silicon technology.

We are happy that the referee identifies the value of the study. The following is a detailed response to the valuable comments and objections that referee #2 has raised.

***

Page 2 line 45: the authors mention that in ABO_3, A = small metal cation, B= large metal cation. This is not a general rule, in fact, for the case study (LNO) the ionic radius of La >> ionic radius of Ni. Many other examples can be mentioned here where A cation is much larger than B cation : $BiFeO_3$, $BaTiO_3$...

We thank the referee for pointing this out. Not only is she/he correct that this is an incorrect generalization, but the statement as it stands is intrinsically wrong. In general, the A cation is of course usually bigger than the B cation for a large range of compounds. Furthermore, such generalizations only serve to cloud our point, and we find that it is best to completely remove it. Again, we thank the referee for noticing this erroneous sentence.

***

What is the role of the precursor chemistry on favoring epitaxial growth? why the use of $Ni(acac)_2$ promotes epitaxy over $Ni(thd)_2$? Would the use of $La(acac)_3$ further improve the growth? The authors should comment on how this effect the growth mechanism.

This is an important comment, and one that we answered in detail in a similar comment from referee 1. We turn this reviewers attention to that answer for a general comment. As an additional comment, we do not believe that $La(acac)_3$ will enhance growth, as a polymerization of the type found in $Ni(acac)_2$ does not take place in $La(acac)_3$ due to it already being octahedrally coordinated.

***

Did the authors try to post anneal the samples and study if they are still strained? It could be good to know if this post thermal treatment changes the structural defect scenario and thus the resistivity behavior.

This is a valid and interesting point. In fact, tuning post-deposition treatment to tune strain and oxygen content is an interesting possibility. The advantage with the reported growth is that post-deposition

annealing can be tuned from the deposition temperature and upwards, with no initial high temperature needed. We have previously briefly studied the effect of annealing on these samples, and we have now revisited this to answer the question raised by the reviewer.

The figure below shows specular XRD for as deposited films and films annealed at 650 ^{o}C in air, on $SrTiO_3$ substrates.

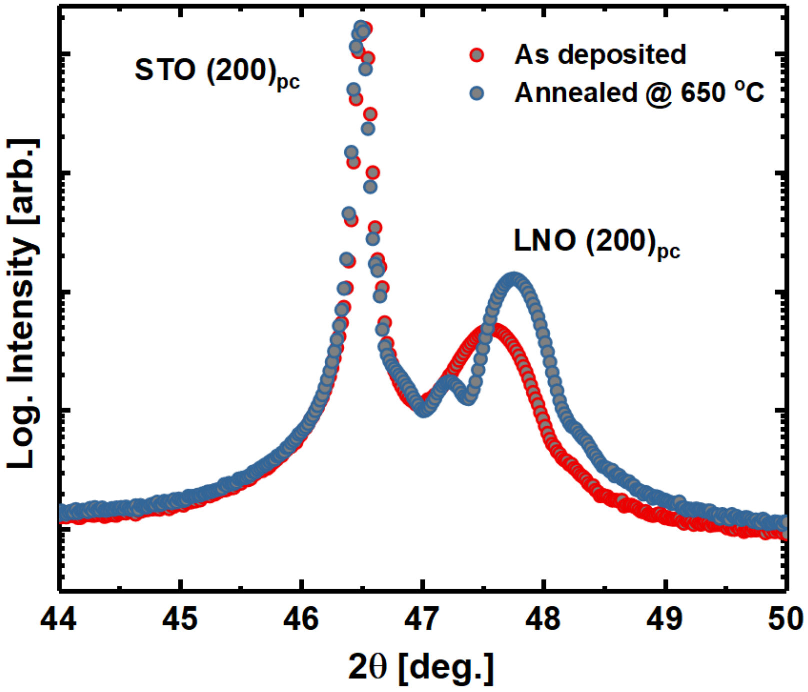

As can be observed, there is a slight shift (approx. 0.2 deg) towards a smaller c-axis, which does indicate some relaxation. In addition, there are more obvious crystal truncation rod features, indicating a somewhat sharper interface towards the substrate, and/or a sharper surface. The overall intensity of the reflection is similar, indicating little extra crystallization taking place upon annealing. Furthermore, we have measured in-plane resistivity of the post annealed sample and found that there is a slight decrease in resistivity, but on the same magnitude as for the as deposited samples (lowest measured resistivity 70 μΩ cm).

While this is indeed very interesting, and something that should be studied more to understand the effects of post deposition treatment on structure and functionality, we believe that the main focus of this paper is the successful deposition of $LaNiO_3$ at low temperature with no post deposition annealing needed.

We do think, however, that it is of the interest to the community to be aware of these findings, and we have included the figure above in the supporting information, together with a paragraph and a sentence in the manuscript that primes these data:

On page 8:

Specular XRD of select samples on $SrTiO_3$ (100) annealed at 650 °C in air was carried out to investigate any relaxation of the imposed strain that was observed for the as-deposited samples (supporting information, Figure E). While the thin film c-axis was slightly compressed upon annealing ($\Delta 2\theta \approx +0.2°$, $\Delta c \approx -0.013$ Å), very little additional crystallization was observed. Some additional fractional fringes are observed, indicative of a somewhat sharper interface and/or surface. Tuning the strain of the as deposited epitaxial films by post deposition annealing is an intriguing possibility, but one that is left for later investigation.

On page 11:

Note that post deposition annealing of these samples provides very little added functionality, with lowest measured resistivity of 70 μΩ cm.

We thank the referee again for this useful comment, and hope the added discussion helps shed light on these issues.

***

It is reported that at very thin films (ultrathin), some materials show a "dead-layer" and they are insulating. Did the authors study which is the minimum thickness by which the samples start to be conductive? That is interesting to know to use the minimum material in monolithic device integration.

This is of course a very interesting point, but we find that to answer this question properly we would need another study designed to answer it. The thickness induced MIT is dependent on many features of the film, including strain, stoichiometry, oxygen content *etc.* Previous reports of films deposited by MBE and PLD have shown thickness dependent MITs on approximately 4 u.c. films. In our studies on $LaNiO_3$ on $LaAlO_3$ we find an upturn in the resistivity starting at films with thickness of 2.5 nm. Films are more or less conductive even at 1.5 nm (approx. 4 u.c., specific resistivity ~1 mΩ cm), but definitely does not conduct as well as the slightly thicker films. A temperature and thickness dependent study (preferably connected with surface diffraction studies) of the resistivity would be needed for a stringent determination of any MIT, but this is out of the scope of this paper. However, we can without a doubt say that the films conduct like metals at least down to 2.5 nm. Since this, as the referee says, is interesting for monolithic device integration, we have added a sentence that states the certain interval of metallic conduction on page 12:

Note that films down to a thickness of 2.5 nm has been characterized and found to conduct with similar resistivity as the 30 nm films that are presented here.

We thank the reviewer for this comment and hope that this provides the necessary information to make the films interesting in the scope of monolithic device integration.

***

Clearly ALD is a technique to prepare thin films and 30 nm is an "appropriate" thickness but, up to which thickness these ALD films are strained?

This is again a very interesting question, but unfortunately also the only question where we cannot provide an answer. We have deposited films successfully up to slightly above 60 nm (corresponding to 2000 ALD-cycles), and at that stage we are starting to worry about depletion of our $Ni(acac)_2$ source. This would create unwanted chemical inhomogeneity in the samples that would cloud any useful information. At 60 nm the films (not surprisingly) carry the same strain as the 30 nm films. At some thickness the films will of course relax, but ALD is not the technique to investigate these matters. We are sorry that we are not able to provide a more clear answer on this matter.

No action was taken.

***

Reviewer #3

I have carefully read the manuscript by Dr. Sønsteby and colleagues, and I believe it belongs in a more specialized journal.

We are grateful that the referee has found the time to carefully read the manuscript, even if she/he finds that it belongs in a more specialized journal. Given the positive comments on impact and novelty from reviewers 1 and 2, in addition to our new revisions and comments to reviewer 3, we hope to convince her/him that the work carries the necessary impact to warrant publication in Nature Communications.

***

The authors report a low temperature ALD growth of $LaNiO_3$ on $SrTiO_3$ and $LaAlO_3$ substrates. By itself, the ALD growth of perovskites is not particularly new. In particularly, LNO growth on STO has been recently reported by Ritala et al. What sets this work apart is crystallization upon deposition at 225C that eliminates the need for crystallization anneal (typically about 700C). And one could argue that this choice of precursors and oxidizer, resulting in low temperature process, makes it compatible with the back of the line (BOL) processing in Si CMOS. However, the authors are focusing on all oxide electronics, where this is not even an issue.

While the referee is correct that ALD of $LaNiO_3$ has been reported before, this is not really the novelty of our work. As we have stated in the paper, previously reported routes for deposition of LNO has involved a much higher thermal budget, which is not compatible with most device integration schemes. The referee has noted that the novelty lies in the low temperature epitaxy, and we believe this carries wide impact. While we focus on all oxide electronics, there is nothing to say that epitaxial integration on *e.g.* STO-buffered or SrO-terminated Si is not attainable. We provide a platform for low-T epitaxy of a metallic perovskite, and do not rule out any application.

The paper is full of strong statements, such as that "oxide electronics is rising fast as a contender to Si-based circuitry," or that the integration of LNO on STO is "a highly waited example in technology industry." At best, these are artistic exaggerations.

We thank the referee for this comment and have tried to tone down the strong statements mentioned by the referee.

"…oxide electronics is rising fast as a contender to Si-based circuitry, …", now reads "…oxide electronics is considered a viable future contender to Si-based circuitry, …"

"a highly awaited example in technology industry" now reads "a technologically interesting example"

Furthermore, we have changed four other statements that we have identified to be too strong:

On page 4:
"Although ALD traditionally have been used to deposit binary oxides, heavier chalcogenides and halides, there has been a dramatic increase in reports of functional complex oxides over the last 10 years." now reads: "Although ALD traditionally have been used to deposit binary oxides, heavier chalcogenides and halides, there has been an increase in reports of functional complex oxides over the last 10 years."

On page 5:

"No post annealing is required to obtain the highly regarded metallic properties." now reads: "No post annealing is required to obtain metallicity."

On page 5:

"It fundamentally extends the toolbox of available low-temperature deposited epitaxial complex oxides with appreciable functional properties." now reads: "It extends the toolbox of available low-temperature deposited epitaxial complex oxides with appreciable functional properties.".

On page 14:

"This new process maintains an extremely low thermal budget that should be compatible with most device fabrication, and especially in monolithic device integration in transistor technology." now reads: "This new process maintains a low thermal budget that should be compatible with most device fabrication, and especially in monolithic device integration in transistor technology."

To me, the paper shows low temperature crystallization (it does occasionally happen in ALD, for example BaTiO3 on STO or even on Ge (001)) of LNO on STO and LAO. The films are partially relaxed, and show expected electrical behavior. The crystal quality is similar to that of a typical ALD film (plus some porosity). This is another process in the tool box of oxide electronics that has been hyped since mid-nineties and is yet to show some technological promise.

We thank the reviewer for these thoughts, but we do not think that "low-temperature crystallization" is the correct way to describe this process. If LNO did in fact crystallize at these low temperatures, this would also happen with different precursor chemistry. We have gone a long way in providing evidence that the precursor chemistry is very important here, and low temperature epitaxy is not attainable with other reported routes for depositing LNO with ALD. Furthermore, while the films show electrical behavior similar to that of bulk LNO, the novelty here does not lie in very low resistivity, but obtaining these properties at low deposition temperatures. We are aware of growth of BTO on STO and Ge(001), and highly appreciate these results. However, the reviewer will certainly be aware that the crystallinity of BTO films on STO is significantly improved upon annealing, and that the growth of BTO on Ge is only attainable as homoepitaxy after depositing a buffer layer of BTO first.

What we provide here is a low temperature route for an as deposited epitaxial perovskite with metallic properties, which is important wherever a conducting perovskite template material is required. To the best of our knowledge, no such route has been identified previously, regardless of technique employed. We are

quite certain that the impact of this is within the scope of Nature Communications, particularly when considering the large amount of academic and industrial research on this topic.

We do hope and believe that we have been able to convince reviewer 3 that this work carries significant impact.

PS! In addition to the referee's comments, some small typographical changes have been made. These have been highlighted in the manuscript. Also note that the title of the y-axis in figure 9 has been changed to μΩ cm instead of the erroneous Ω cm.

Reviewers' comments:

Reviewer #2 (Remarks to the Author):

The revisions to this manuscript are mostly sufficient. In this second review, I have a few additional points concerning the implied (but false) novelty of partial coherence in electron ptychography (apologies to the authors for not catching this in my first review but I think it is important enough) and one further comment left over from my first review. Provided that the implied additional revisions are addressed with sufficient rigor, I would recommend publication.

1. Manuscript states "High coherent field emission guns are widely used as the electron sources in modern electron microscopes and so electron ptychography usually assumes only a pure coherent state of the illumination probe19,26,27." And later the manuscript states "There are only a few proof-of-principle demonstrations of mixed-state electron ptychography28,29 and while these suggest the promise of the approach, to date no high-quality atomic resolution or sub-angstrom resolution reconstructions have been achieved."

The effects of partial coherence (mixed state) were explored in ref 18, and the effects were already well understood in electron ptychography, and more generally x-ray and electron phase retrieval, well before then. Ref 18 achieved a spatial resolution of about 1 Angstrom (this is "atomic resolution"). Ref 18 achieved reconstructions of high quality. One point of difference, however, is that in ref 18 partial coherence did not so dramatically affect the results, that is, assuming perfect coherence did not produce "completely wrong" reconstructions (nor should it). This is in contrast with the present manuscript where "completely wrong" reconstructions ARE seen - a result which is very surprising and not in itself comprehendible (as I wrote in my first review). In summary, the authors should revise the statements in their manuscript so that they more directly acknowledge the fact that partial coherence (or mixed state, different name for same thing) in electron ptychography is already well-known and well-understood from previous works.

2. Manuscript states "Similar setups with a defocused probe have been adopted previously17,18,26 to overcome the slow readout speed of conventional CCD cameras and limited stability of the imaging systems."

This statement is not correct. Yes, similar (essentially identical) setups HAVE been used but the probe was not defocused for the reasons stated by the authors, it was defocused as an essential part of the ptychographic scheme (and the authors have done the same thing).

3. First review: "A mixed-state model of the electron probe ...is able to account many of the different factors ... such as the finite electron source size, chromatic aberration, point spread function of the detector, sample vibration, and fast instabilities of the image-forming system" Should the detector PSF be included in this list? Finite source size (sum of incoherent probes) does reduce the interference between different diffraction paths, but the sharpness of the diffraction discs remains the same.

Authors' response: The detector PSF degrades the sharpness of the diffraction disc and reduces the coherence, details can be found in Ref. [23] and [24]. We have already included those references. The incoherence from the finite source size comes from the incoherent sum of diffractions from different spatial positions. Such incoherent summation results in reduced contrast of the coherent speckles due to the different phase change of the probe wave function induced by the sample. Therefore, finite source size can be naturally covered within mixed-state model. A good discussion has been given in Ref. [24].

The authors have not understood my point (and admittedly I did not write it very clearly). The detector PSF DOES blur the diffraction discs but the assumed mixed state model DOES NOT (it only reduces the interference between diffraction discs). So, the detector PSF produces effects which are not included by the mixed state model. So, the authors should revise their statement.
